# Transcriptomics reveal core activities of the plant growth-promoting bacterium *Delftia acidovorans* RAY209 during interaction with canola and soybean roots

**DOI:** 10.1099/mgen.0.000462

**Published:** 2020-11-05

**Authors:** Danae M. Suchan, Jordyn Bergsveinson, Lori Manzon, Alexa Pierce, Yuriy Kryachko, Darren Korber, Yifang Tan, Dinah D. Tambalo, Nurul H. Khan, Michael Whiting, Christopher K. Yost

**Affiliations:** ^1^​ Department of Biology, University of Regina, Regina, SK, Canada; ^2^​ Lallemand Plant Care North America, Saskatoon, SK, Canada; ^3^​ Department of Food and Bioproduct Sciences, University of Saskatchewan, Saskatoon, SK, Canada; ^4^​ Aquatic Crop Resource Development, National Research Council, Saskatoon, SK, Canada

**Keywords:** canola, *Delftia*, inoculant, PGPR, RNA-seq, soybean

## Abstract

The plant growth-promoting rhizobacterium *Delftia acidovorans* RAY209 is capable of establishing strong root attachment during early plant development at 7 days post-inoculation. The transcriptional response of RAY209 was measured using RNA-seq during early (day 2) and sustained (day 7) root colonization of canola plants, capturing RAY209 differentiation from a medium-suspended cell state to a strongly root-attached cell state. Transcriptomic data was collected in an identical manner during RAY209 interaction with soybean roots to explore the putative root colonization response to this globally relevant crop. Analysis indicated there is an increased number of significantly differentially expressed genes between medium-suspended and root-attached cells during early soybean root colonization relative to sustained colonization, while the opposite temporal pattern was observed for canola root colonization. Regardless of the plant host, root-attached RAY209 cells exhibited the least amount of differential gene expression between early and sustained root colonization. Root-attached cells of either canola or soybean roots expressed high levels of a fasciclin gene homolog encoding an adhesion protein, as well as genes encoding hydrolases, multiple biosynthetic processes, and membrane transport. Notably, while RAY209 ABC transporter genes of similar function were transcribed during attachment to either canola or soybean roots, several transporter genes were uniquely differentially expressed during colonization of the respective plant hosts. In turn, both canola and soybean plants expressed genes encoding pectin lyase and hydrolases – enzymes with purported function in remodelling extracellular matrices in response to RAY209 colonization. RAY209 exhibited both a core regulatory response and a planthost-specific regulatory response to root colonization, indicating that RAY209 specifically adjusts its cellular activities to adapt to the canola and soybean root environments. This transcriptomic data defines the basic RAY209 response as both a canola and soybean commercial crop and seed inoculant.

## Data Summary

Raw sequencing reads have been deposited into National Center for Biotechnology Information – Sequence Read Archive (SRA) under number PRJNA544929, with accession numbers SAMN11866859–SAMN11866882.

**Impact Statement**

It is well established that plant growth can benefit from interactions with advantageous bacteria in the soil. To attract and maintain a beneficial root-associated microbial community, plants can exude 20–30 % of all carbon captured by photosynthesis into the rhizosphere, making the plant root an ideal target for bacterial colonization. Arguably, a critical component of the plant–microbe interaction is the ability of the bacterium to colonize the roots of the plant host. The dynamic nature of the plant-root environment and the physiological changes throughout plant maturation present significant challenges to bacterial colonization, especially considering that these changes and developments can vary substantially among plant taxa. This research investigated the regulatory networks of gene expression (RNA-seq) transcriptional analysis to study the root colonization of a plant growth-promoting bacterium *Delftia acidovorans* RAY209 in the developing root systems of the two largest oil-seed crops grown globally, canola and soybean. Just as the benefits conferred by a plant growth-promoting bacterium may be distinct between different plants, it was hypothesized that RAY209 would exhibit distinct transcriptional strategies within two distinct root environments, and that identifying these differences would provide novel insights into root association and colonization mechanisms by plant-associated bacteria.

## Introduction

To attract and maintain a beneficial root microbiome, plants can exude 20–30 % of photosynthate into the rhizosphere, making the plant root an ideal target for bacterial colonization [1–3]. Several agriculturally relevant rhizobacteria have been shown to establish root colonization through a two-phase process: an initial weak, transient and reversible root association is followed by the distinct transition to a strong and irreversible root attachment through the production of various extracellular components required for bacterial cell anchoring and aggregation [4]. Once root-attached, specific members of a plant’s root microbiome have the capacity to promote plant growth through a variety of mechanisms, including bacterial production of phytohormones, production of iron siderophores that can mobilize iron to the plant, nitrogen fixation, and antagonism of plant pathogen growth [5]. Advancements in molecular and genomic technologies have improved the mechanistic understanding of some of these interactions between plant growth-promoting rhizobacteria (PGPRs) and plant hosts, and have highlighted their importance in understanding overall rhizosphere ecology and plant health.

The global demand for increased crop vigour and yield has stimulated interest in the commercial development of PGPR inoculants for improved crop growth, creating the need for a deeper understanding of PGPR activity [6]. Such inoculants, which contain either a singular isolate or a mixture of a few distinct PGPR isolates, are traditionally used for pulse crops due to the known benefits of symbiotic plant–microbe nitrogen fixation [7]. However, the development and appropriate application of PGPR inoculants for other plant crop species may provide a tool for farmers to address the global challenge of increasing food production without increasing negative impacts to environmental systems [8, 9].

PGPR inoculant activity is generally preceded by successful plant host root attachment, followed by established colonization [4, 10]. However, the dynamic nature of the plant-root environment and the physiological changes throughout maturation present significant challenges to bacterial colonization, especially considering that these changes and developments can vary substantially among plant taxa [1, 11, 12]. Further contributing to the complexity of plant–microbe interactions is the assumption that the genomic capacity of a specific bacterium governs its ability to adapt to and successfully colonize a given root. Thus, investigation of PGPR gene regulation during early and sustained root colonization is a straightforward approach to help identify genetic traits and regulatory responses that confer adaptations or advantages for root colonization.

The betaproteobacteria *Delftia acidovorans* RAY209 (RAY209) was previously isolated from a canola rhizosphere growing in Western Canadian soil, and the genome sequence was recently published [13]. RAY209 is registered by the Canadian Food Inspection Agency under the *Fertilisers Act and Regulations* for use as a PGPR inoculant for two globally important crop plants, canola (*Brassica rapa* subsp. *oleifera*) and soybean (*Glycine max*). The respective global production of canola and soybean has been reported to be up to 68 million and 337 million metric tonnes [14], with crop production increasing in response to rising consumption and demand. Therefore, investigation of the genetic mechanisms that enable RAY209 to colonize the roots of these important crops will help facilitate the selection of other PGPR inoculants, and can equip crop producers with an empirical basis of understanding for optimal RAY209 application.

This study uses RNA-seq transcriptional analysis to investigate the functional activities of RAY209 during interaction with the developing root systems of canola and soybean plants. Extensive experimentation was performed with the canola root environment to first confirm that RAY209 is capable of exhibiting a two-phase root colonization response, then to validate a method for differential collection of the irreversibly root-attached cells from the reversibly root-associated cells [4]. We posit that RAY209 collected from plant roots using this validated method constitutes cells exhibiting a root colonization response, although the soybean root colonization described in this study is to be considered putative based on a lack of direct physical confirmation. Just as the benefits conferred by a PGPR inoculant may be distinct between different plants, it is hypothesized that RAY209 will exhibit distinct transcriptional responses to the colonization of two unique root environments, and that identifying these differences will provide novel insights into root colonization mechanisms by rhizobacteria.

## Methods

### Canola and soybean hydroponic growth systems

Canola and soybean plants were prepared and cultivated independently, with both types of seeds first sterilized with 2 % NaClO and rinsed with sterile water. Seeds were germinated in the dark on sterile polystyrene mesh squares set atop 0.2 % agar for 4 days. *D. acidovorans* RAY209 was grown overnight on solid lysogeny broth (LB) medium with 0.5% NaCl, and subsequently inoculated into tryptic soy broth (TSB) and grown overnight at 30 °C with shaking at 200 r.p.m. On the fourth day of seed germination, overnight growth of RAY209 was prepared to a volume supporting an inoculation concentration of 5.5×10^7^ c.f.u. ml^−1^, with 0.5× Hoagland’s solution (Sigma-Aldrich) as diluent. This volume was applied to the bottom of a sterile box, and then mesh screens with sprouted canola or soybean were removed from the germination agar and set on the surface edge of the box (experimental day 0). These sacrificial plant growth box systems were prepared in triplicate for each experimental time point, alongside negative control boxes of ‘plant, no RAY209’ and ‘no plant, RAY209’. All plant growth box systems were incubated at room temperature within a biosafety cabinet with 16 h light exposure.

Plant growth box systems were monitored daily, with the liquid medium level maintained at the level of the mesh supporting the plant roots by ‘watering’ with 0.5× Hoagland’s solution every 2 days. RAY209 plating and cell count determination were performed to monitor survival and detect contamination by collecting and combining 200 µl from each corner of the plant growth box, vortexing briefly and serial diluting in 1X PBS solution. Dilutions of cell suspensions were plated on tryptic soy agar (TSA) and incubated at 30 °C overnight.

To ensure adequate oxygenation within the liquid plant growth medium, dissolved oxygen concentrations were measured at canola roots with a YSI meter prior to root harvesting on day 2 and day 7 (Fig. S1). Between each measurement, the probe was sterilized in a 2% NaClO solution for 3 min and rinsed with sterile water.

### Determination of RAY209 colonization of canola

**Fig. 1.**
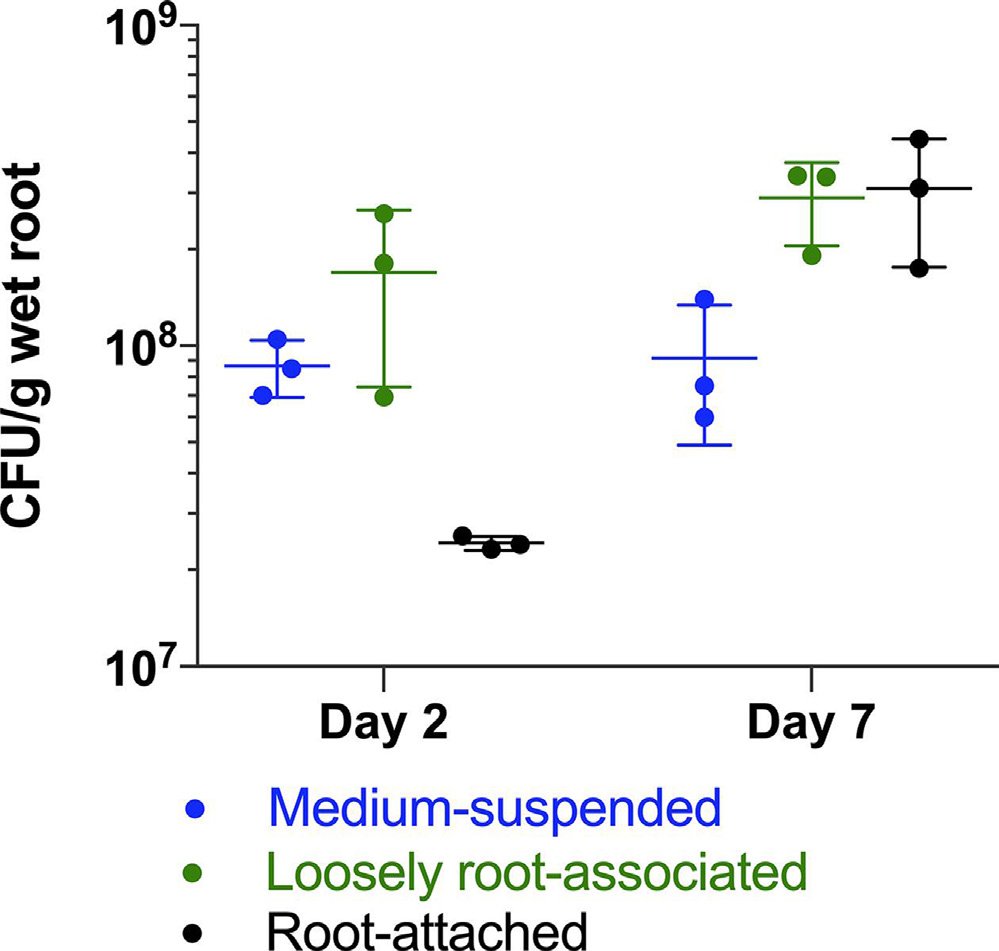
Cell counts of medium-suspended and canola root-colonizing RAY209. Medium-suspended cell numbers were stable over the 7 day growth period in the hydroponic canola root boxes (*P*_two tail_ 0.91). While the loosely root-associated cell numbers also remained constant from day 2 to day 7 (*P*_two tail_ 0.18), there was a statistically significant log_10_-fold increase in the root-attached cell numbers over this time period (*P*_one tail_ 0.03). *n*=3 replicates. The corresponding day 7 RNA-seq dataset specifically isolated this root-attached RAY209 population for insight into root colonization regulatory gene networks.

**Fig. 2.**
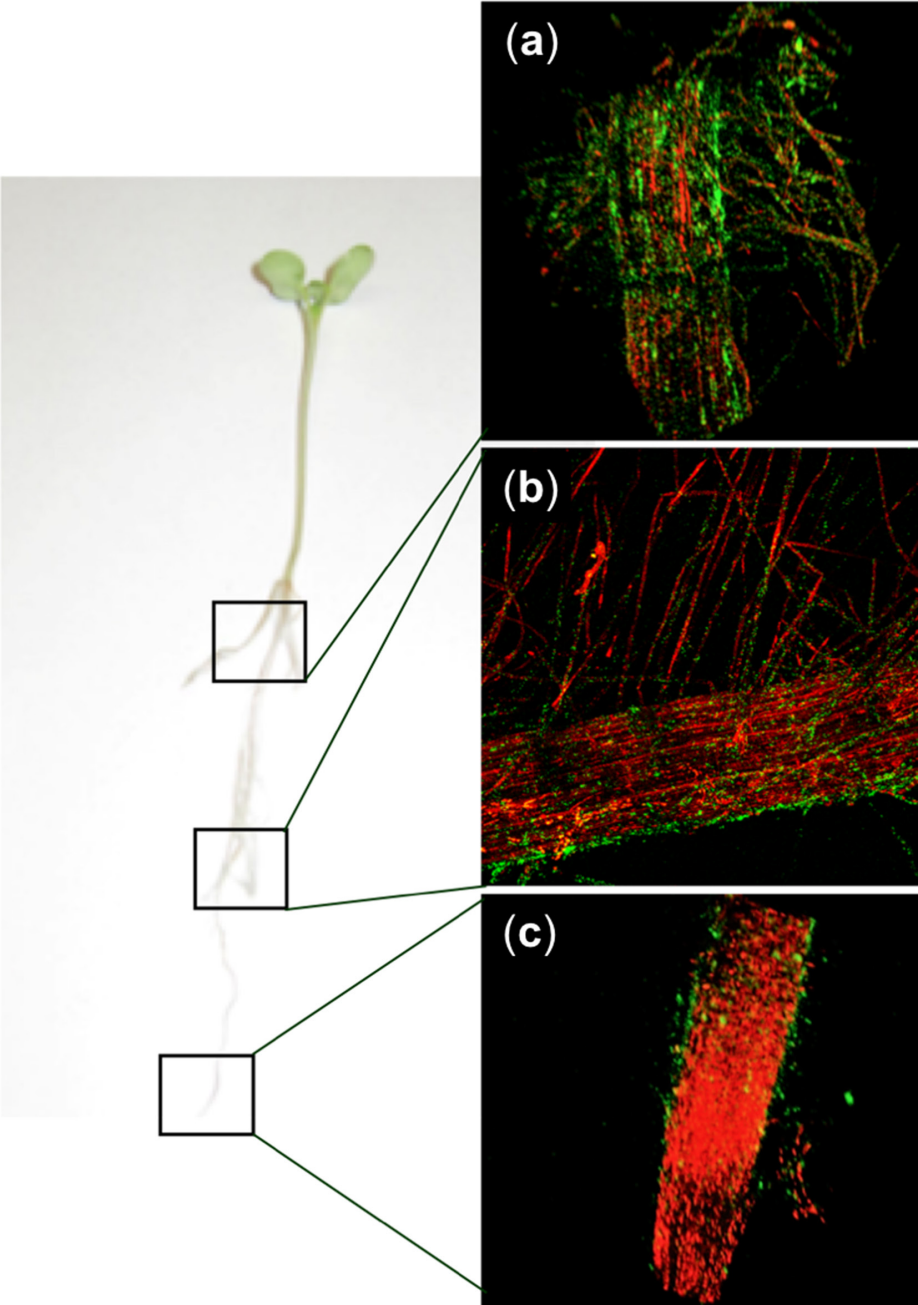
Colonization of Nile red-stained canola roots with 1.0×10^5^ c.f.u. of *D. acidovorans* RAY209-GFP per seed. Magnification ×10, 2-channel image. (a) Canola root adjacent to stem; (b) middle of root; (c) root tip. Coverage of the entire root surface [a+b+c] by RAY209-GFP was 40.33±8.39 % (*n*=3 for each area).

RAY209 root colonization and PGPR activities were confirmed on canola plant roots through multiple means of assessment, including RAY209 root attachment assays (Figs 1, S2 and S3, available with the online version of this article), confocal microscopy visualization of GFP-labelled RAY209 colonizing canola root surfaces (Fig. 2), and an examination of plant traits (Fig. S4, Table S1). A root attachment assay was conducted with canola plants to confirm the presence of an irreversibly root-attached RAY209 population by day 7 (Fig. 1). Cell counts were performed at day 2 and day 7, and were normalized by the wet root mass input for each sample. Medium-suspended cells were compositely sampled by pooling 200 µl volumes from each corner of a given hydroponic box and vortexing to mix. Root samples were collected by removing mesh screens with plant roots from each box and using a sterile scalpel to cut roots on the underside of the mesh from the point where they were submerged in liquid medium, and were then transferred to separate Falcon tubes to record the wet root masses for normalization. Loosely root-associated cells were dislodged by adding 10 ml 1X PBS to each tube of roots and vortexing gently on medium speed for 10 s. The loosely root-associated cells were sampled from the supernatant, and the roots from each replicate were transferred into separate tubes to process the strongly root-attached RAY209 cells. Roots were manually homogenized for 5 min with a tissue grinder, vortexed at maximum speed for 30 s and gently spun down by centrifuging at 1250 ***g*** for 30 s. Cells were sampled from the liquid released by the roots during the homogenization process, which was accounted for in the wet root mass normalization. The numbers of c.f.u. were obtained by serially diluting cells with 1X PBS, plating on TSA and incubating overnight at 30 °C.

### Construction of the RAY209-GFP strain

*D. acidovorans* RAY209*-*GFP (RAY209-GFP) strains were obtained via one parental conjugation of RAY209 with *Escherichia coli* S17 λ pir pKN *tfd gfp*. The GFP-containing *E. coli* donor strain, constructed by Ng [15], was kindly provided by Dr Julie Zilles from the University of Illinois at Urbana-Champaign (Urbana-Champaign, IL, USA). Transconjugant GFP-positive RAY209 strains were visually selected as green-fluorescing colonies on kanamycin-containing (50 µg ml^−1^) TSA plates under long-wave UV illumination. The brightest transconjugate strain, *Delftia* sp. RAY209-GFP 2–2 (RAY209-GFP), was selected for root colonization experiments.

### Colonization and staining of canola roots

Three surface-sterilized canola seeds were placed in CYG germination pouches (Mega International) and 10 µl RAY209-GFP (standardized to reach the desired c.f.u. per seed concentration), or 10 µl sterile dH_2_O for control plants, added to the back of the pouch (day 0). This was performed to assess motility of RAY209 to the canola seeds. For effective germination and to maintain sterility, all pouches were covered with tinfoil and placed in a phytotron (Conviron PGR15) with a day/night cycle of 16/8 h and 22/18 °C. Tinfoil was removed after 48 h and sterile dH_2_O added to each pouch to maintain the seedlings until imaging (day 7).

For imaging, a Nile red stock solution (Tokyo Chemical Industry) in DMSO (Fisher Scientific) (0.5 g l^−1^) was diluted 50× with distilled water and used as the Nile red working solution (NRWS). Root material separated from a live canola plant was sufficiently covered by NRWS in a sterile plastic Petri dish and stained for 10 min. NRWS was then removed, and the stained root placed and affixed in the centre of the dish using Scotch tape (3M). Distilled water was then added to the dish to sufficiently ensure the root was fully immersed in water.

### Confocal laser scanning microscopy and image analysis

Fluorescence signals from RAY209-GFP and from canola roots stained with Nile red were visualized using a Nikon C2 confocal microscope system configured with a Nikon Eclipse LV100D-U microscope (Nikon Instruments) with water-immersible Nikon Fluor (×10/0.30 NA, ×40/0.80 NA and ×60/1.00 NA) lenses, and laser excitation lines of 488 and 543 nm. Root tips, mid areas and adjacent-to-stem root areas were analysed using confocal laser scanning microscopy (Fig. 2). NIS Elements Viewer software (Nikon Instruments) was used to quantitate green fluorescence signals from RAY209-GFP (*Ng*=mean number of green objects) and red fluorescence signals from the plant roots (*Nr*=mean number of red objects) in the three areas of a root, using three replicates for each root area. Quantitated green fluorescence was calculated as the fraction of total fluorescence $\left[(Ng\sqrt{Ng+Nr})\times 100\right]$ and represents coverage of an entire canola root surface by RAY209-GFP.

### RAY209 cell harvesting for RNA extraction

On day 2 and day 7 following initial setup and inoculation of canola and soybean growth systems, liquid growth medium and plant roots were sampled to collect medium-suspended and root-attached RAY209 cells for RNA extraction. Root samples were collected by removing mesh screens with plant roots from each box and using a sterile scalpel to cut roots on the underside of the mesh from the point where they were submerged in liquid medium. Cut roots were immediately placed into 20 ml of ice-cold 95 % ethanol:5 % acid phenol. These samples were incubated on ice for 5 min and gently agitated on a vortex mixer to remove any excess loosely root-associated RAY209 cells. Roots were then collected and placed into a sterile foil packet, immediately flash-frozen in liquid N_2_ and stored at −80 °C prior to subsequent processing. Medium-suspended cells were sampled compositely by collecting a total of 10 ml cell suspension from four corners of an individual growth system box. Suspensions were centrifuged at 10 000 ***g*** for 10 min at 4 °C, then resuspended in 4 ml ice-cold 95 % ethanol:5 % acid phenol. Resuspended cells were kept on ice for 45 min, followed by centrifugation at 8200 ***g*** for 15 min at room temperature. Cells were resuspended in 1 ml supernatant and transferred to a fresh tube and collected again by centrifugation at 8200 ***g*** for 5 min. The supernatant was decanted, and the cell pellet flash frozen in liquid N_2_ and stored at −80 °C.

### RNA extraction

Frozen roots in tinfoil packets were gently beaten with a spatula to break the roots into smaller pieces. For each root sample, a total of 0.4 g root pieces was transferred into two separate 1.4 ml tubes containing 0.25 g of 1 mm glass beads. Samples were then processed according to Holmes *et al*. [16] and the two separate extraction preps were combined after chloroform:isoamyl alcohol (24 : 1) and 10 % CTAB (*N*-cetyl-*N*,*N*,*N*-trimethylammonium bromide)/0.7 M NaCl extraction. Combined cell extractions were then processed using an RNeasy mini column kit (Qiagen) according to the manufacturer’s instructions and eluted with 30 µl RNase-free water.

Frozen medium-suspended cell pellets were resuspended in 100 µl TE buffer with 50 mg lysozyme ml^−1^ and 20 mg/ml proteinase K (Qiagen). Suspensions were incubated at room temperature for 5 min, with vortexing every 10 s for 2 min, followed by addition of 1 ml pre-heated (65 °C) TRIzol (Qiagen). Suspensions were vortexed on high for 3 min, incubated at room temperature for 5 min, and then subjected to a chloroform/ethanol extraction. The upper phase of this extraction was collected and processed using an RNeasy mini column kit according to manufacturer’s instructions and eluted in 30 µl RNase-free water.

All RNA eluates were DNase-treated with the Turbo DNA-free kit (Thermo-Fisher) according to the manufacturer’s instructions. RNA samples were then checked for RNA contamination via quantitative PCR, by first making cDNA using a SuperScript VILO cDNA synthesis kit (Invitrogen) and using primers specific for *rpoD*, *gyrA* and 16S rRNA genes of RAY209. Root-attached RNA samples were subjected to rRNA removal using probes from a bacterial and plant seed/root Ribo-Zero depletion kit (Illumina) combined at equal concentration. RNA samples extracted from medium-suspended cells were rRNA depleted using the bacterial Ribo-Zero depletion kit (Illumina). All RNA samples were assessed for quality and size distribution pre- and post-rRNA removal via Bioanalyzer trace (RNA 6000 Nano; Agilent),

### RNA sequencing and data processing

All 24 RNA samples were sequenced on one lane of an Illumina HiSeq version 4 platform using paired-end, 125 bp cycles. Raw reads were trimmed using Trimmomatic v.0.38 [17] for quality (phred 33), a minimum length of 100 bp and a sliding window of 10 : 30, and then aligned to the National Center for Biotechnology Information RefSeq RAY209 genome (ASM244305v1) [13] using Bowtie2 v.2.3.4.3 with flags -X 400 and --very-sensitive [18]. Trimmed reads generated from canola or soybean hosts were also aligned using short-reads mapper star v.2.6.1a_08–27 [19] and assembled using StringTie v.1.2.3d [20, 21] using default parameters to Ensembl genomes of *Brassica napus* release 43 and *Glycine max* v2.1, respectively [22]. HTSeq-count v.0.11.2 was used to generate count matrices for all sample alignments [23], then pairwise statistical comparisons made between replicate samples of experimental condition using DESeq2 [24]. Blast2GO was used to perform functional annotation and gene ontology (GO) assignment of significantly differentially expressed (sigDE) (*P*adj<0.05) transcripts [25].

## Results

### RAY209 colonization of canola

*D. acidovorans* RAY209 is commercially formulated as a PGPR inoculant for canola seeds (BioBoost Liquid; Lallemand Plant Care) and is formulated along with *Bradyrhizobium diazoefficiens* USDA 110 as a co-inoculant for soybean seeds (BioBoost+; Lallemand Plant Care). Here, we confirmed the strong attachment and colonization of canola roots by RAY209 (Figs 1, 2, S2 and S3) and its PGPR activity (Fig. S4, Table S1) within a hydroponic growth system (Figs S5 and S6). A canola root attachment assay was done to validate the distinct RAY209 cell populations referred to throughout the study – medium-suspended cells are the planktonic, non-root-associated population; loosely root-associated cells are the weak, reversibly root-interacting population; root-attached cells are the strong, irreversibly attached population anchored to the root (Fig. 1). The medium-suspended and root-attached RAY209 cell populations were examined with RNA-seq in order to capture the differences between these distinct life stages.

At day 2 post-inoculation, RAY209 cells primarily remained medium-suspended or loosely root-associated in a pre-colonization phase (Fig. 1). However, by day 7, a significant log_10_-fold subset of these cells had transitioned to the strongly root-attached state as they successfully colonized the root surface. This population requires rigorous homogenization to be effectively detached from the root, as described in the supplementary material (Fig. S3). Thus, we posit that the RAY209 cells harvested in this manner comprise the irreversibly root-attached population [4] and were treated as such for the purposes of the subsequent RNA-seq experiments and analyses. The same cell harvesting approach was used to evaluate the transcriptional response of RAY209 during interaction with soybean roots and differentiate RAY209’s planthost-specific transcriptional response from its core transcriptional activities employed during exposure to the roots of either plant type.

### Experimental RNA-seq conditions

For the transcriptional experiment, canola and soybean seeds were germinated separately for 4 days, then transferred to grow hydroponically in the presence of 0.5× nutrient-rich Hoagland’s solution (Figs S5 and S6). The hydroponic box systems were inoculated with 5.5×10^7^ c.f.u. *D. acidovorans* RAY209 ml^−1^, and at 2 and 7 days post-inoculation, RAY209 cells designated as medium-suspended (‘M’; present in the liquid hydroponic medium) and root-attached (‘R’; anchored to the root surface) were recovered in triplicate from both plant systems for RNA isolation and subsequent RNA-seq analysis. Read mapping of all 24 samples indicated that between 7 and 8 million reads from the root-attached samples were plant-derived (either canola and/or soybean), leaving roughly 1 million non-16S rRNA reads mapping to RAY209 root-attached cells (Table S2). This provided sufficient sequencing depth for statistical identification of the sigDE (*P*adj <0.05) genes between medium-suspended and root-attached RAY209 (Figs S7 and S8), as well as the opportunity to partially profile the transcriptional response of the respective plant host root tissues to RAY209 colonization.

This experimental design afforded the opportunity to compare the transcriptional profiles of medium-suspended and root-attached cell states on specific plant hosts under several conditions. These included temporal comparisons of RAY209 populations during early and sustained colonization states, as well as during interaction with different plant host root environments. Subsequent analysis of sigDE genes was restricted to those that exhibited greater than or equal to a fourfold (2 log_2_) change in transcript abundance.

### Early versus sustained root colonization

The greatest number of sigDE RAY209 genes occurred between medium-suspended and root-attached cell states during early colonization of soybean (i.e. M D2 vs R D2; 823 sigDE genes; Table 1) and sustained colonization of canola (i.e. M D7 vs R D7; 847 sigDE genes; Table 1). There are considerably fewer sigDE genes between early and late root-attached cells for each plant type [i.e. canola R D2 vs R D7 (206 sigDE); soybean R D2 vs R D7 (70 sigDE); Table 1]. The vast difference in the number of sigDE genes involved in the two processes indicates that a more extensive transcriptional response is required for medium-suspended RAY209 cells to transition to the root-attached state than is required for the root-attached cell population to maintain colonization over time. This in turn could suggest that the initial phases of root attachment pose a greater environmental challenge than maintaining root colonization on a plant undergoing physiological maturations.

**Table 1.** Number of sigDE genes between medium-suspended and root-attached RAY209 cells on canola and soybean

Plant	Comparison (condition 1 vs condition 2)	Total sigDE genes (*P*adj <0.05)	No. of genes increased in condition 1	No. of genes increased in condition 2
Canola	M D2 vs R D2	594	293	301
	M D7 vs R D7	847	432	415
	R D2 vs RD7	206	132	74
Soybean	M D2 vs R D2	823	370	453
	M D7 vs R D7	447	141	306
	R D2 vs R D7	70	33	37

D2, Day 2; D7, day 7; M, medium-suspended; R, root-attached.

**Fig. 3.**
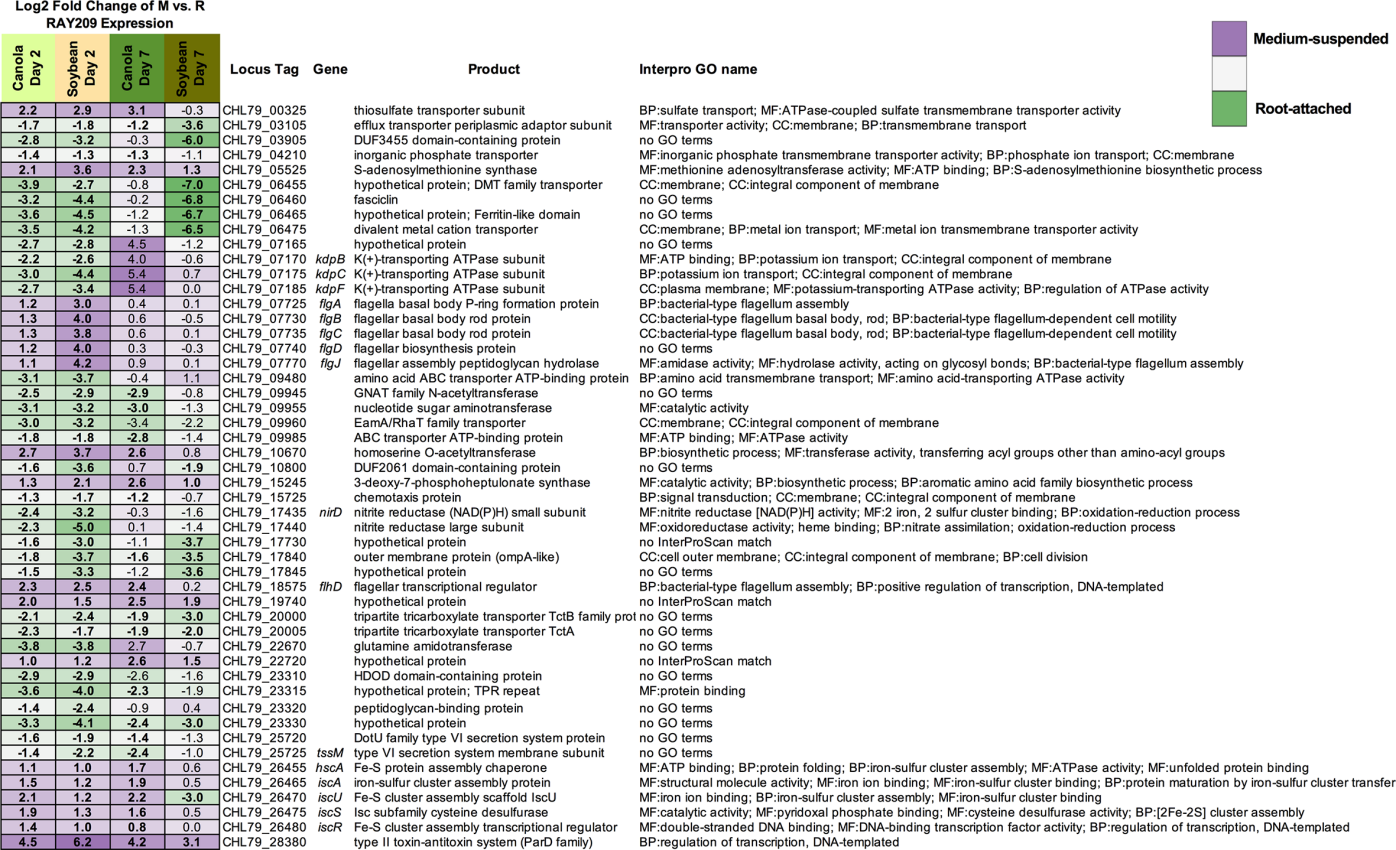
Fifty representative sigDE RAY209 genes between medium-suspended (M) and root-attached (R) states common in canola and soybean. Purple shading indicates increased expression in medium-suspended cells; green shading indicates increased expression in root-attached cells. Bold entries indicate expression is sigDE (*P*adj <0.05). InterPro GO names provide the functional annotation category: BP, biological process; CC,cellular component; MF, molecular function.

The expression profiles of medium-suspended and root-attached RAY209 cells are somewhat conserved between plant types, illustrated by 173 shared sigDE genes during early colonization (D2) and 134 during sustained colonization (D7) (Table 2). These genes likely represent the core colonization response induced in RAY209 as medium-suspended cells navigate and adhere to the roots of either plant, and transition to the strongly root-attached state as they establish sustained colonization between day 2 and day 7 (Fig. 3). Notably, genes related to motility (flagellar assembly; CHL79_07725–CHL79_07770), energy production and iron-sulfur storage (CHL79_26455; CHL79_26470–CHL79_26480) exhibited increased expression in medium-suspended cells (Fig. 3). The strong and directed metabolic response elicited from RAY209 cells suspended in the relatively nutrient-depleted plant growth medium provides insight into the induced chemotactic response that prompts RAY209 to seek the nutrient-replete plant-root environment provided by root exudation. Genes for amino acid biosynthesis (CHL79_05525; CHL79_15245; CHL79_26475) were also up-regulated in the medium-suspended cell population (Fig. 3), which further supports that these cells are experiencing nutrient-deficient conditions. In turn, a fasciclin-like gene (CHL79_06460), encoding a cell-surface protein related to cellular adhesion in plants and other eukaryotes, was among the most strongly up-regulated transcripts in root-attached cells relative to medium-suspended cells of both plant types, implying a significant transition to strong attachment or adhesion of RAY209 to either plant-root system (Fig. 3). This is supported by the log_10_-fold increase in root-attached cells observed between days 2 and 7 of the RAY209 canola root colonization assay (Fig. 1).

**Table 2.** Number of shared sigDE RAY209 genes for comparable experimental conditions between and within plant types

Condition comparison 1*	No. of shared sigDE genes (*P*adj <0.05)	Condition comparison 2*
**D2** Canola M vs R (594 sigDE)	295	**D7** Canola M vs R (847 sigDE)
**D2** Soybean M vs R (823 sigDE)	163	**D7** Soybean M vs R (447 sigDE)
**D2** Canola M vs R (594 sigDE)	173	**D2** Soybean M vs R (823 sigDE)
**D7** Canola M vs R (847 sigDE)	134	**D7** Soybean M vs R (447 sigDE)
**D2** Canola R vs soybean R (501 sigDE)	132	**D7** Canola R vs soybean R (276 sigDE)

D2, Day 2; D7, day 7; M, medium-suspended; R, root-attached.

*Total number of sigDE gene for a given comparison are provided in parentheses.

Several specific RAY209 transcripts are uniquely differentially expressed during growth in the presence of either canola or soybean (Fig. 3), including CHL79_03105 (efflux transporter component) and CHL79_26470 (*iscU*; FE-S cluster assembly scaffold). Differences in magnitude and/or direction of fold-change for specific transcripts across conditions underlines the dynamic behaviour required of root-colonizing bacteria and suggests potential modifications in PGPR gene expression elicited in response to plant-specific root environmental conditions [26].

### Core RAY209 transcriptome response during interaction with canola and soybean roots

Temporal comparison of root-attached RAY209 transcriptional response to growth on canola and soybean reveals 501 shared sigDE transcripts during the early colonization response (D2), counter to 276 shared sigDE transcripts after sustained colonization (D7) (Table 2). This suggests that RAY209 mounts a larger core transcriptional response during the initial phases of root attachment compared to the later phase of sustained root colonization. Shared amongst these expression profiles are 132 RAY209 transcripts that consistently experience increased sigDE when root-attached with both canola and soybean during both early (D2) and sustained (D7) colonization (Table 2).

**Fig. 4.**
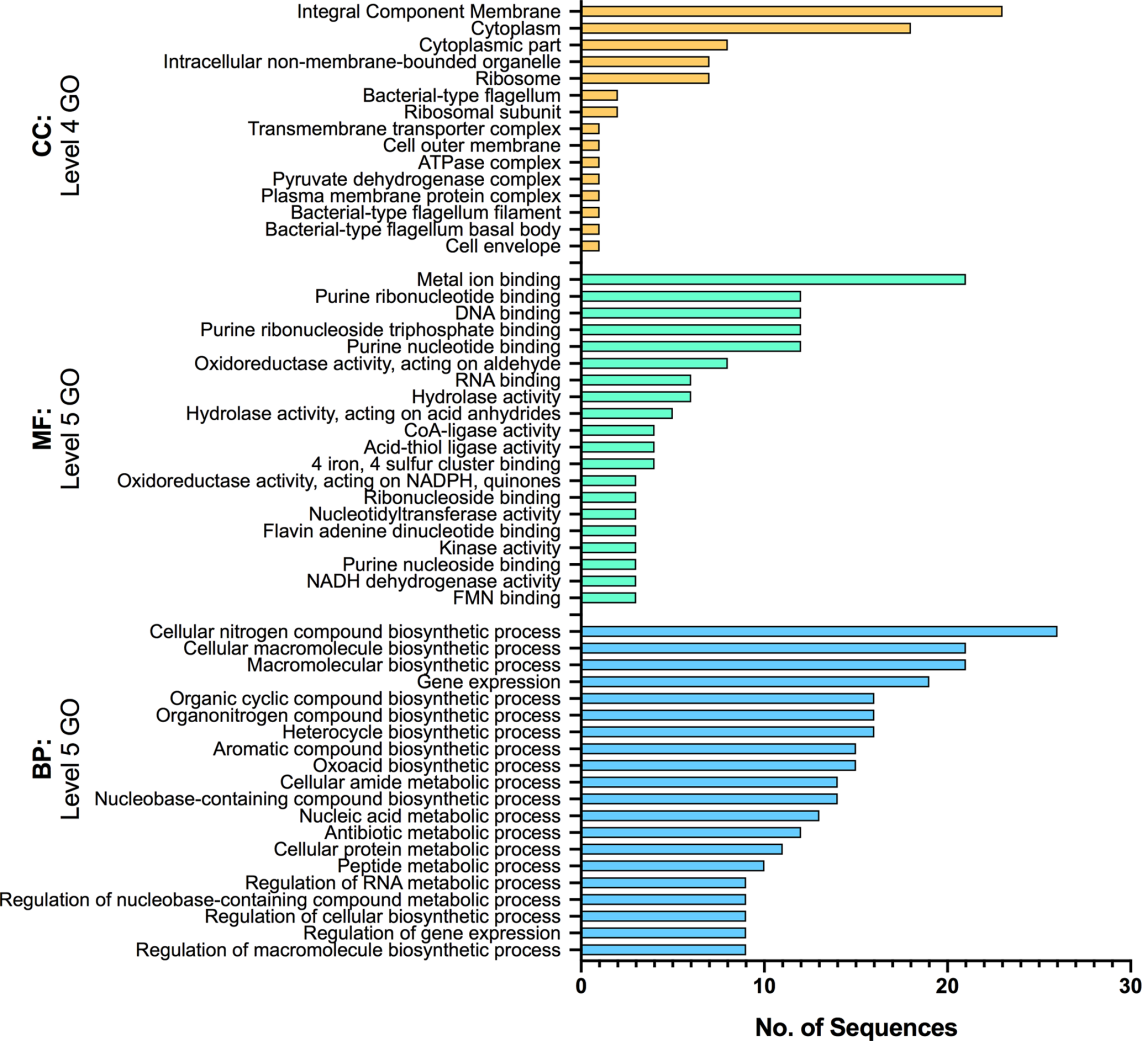
GO annotations of 132 core genes experiencing differential expression during early and sustained association with both canola and soybean. The GO terms provide the functional annotation category: BP, biological process; CC, cellular component; MF, molecular function. The levels indicate the specificity of annotation. SigDE *P*adj <0.05.

**Fig. 5.**
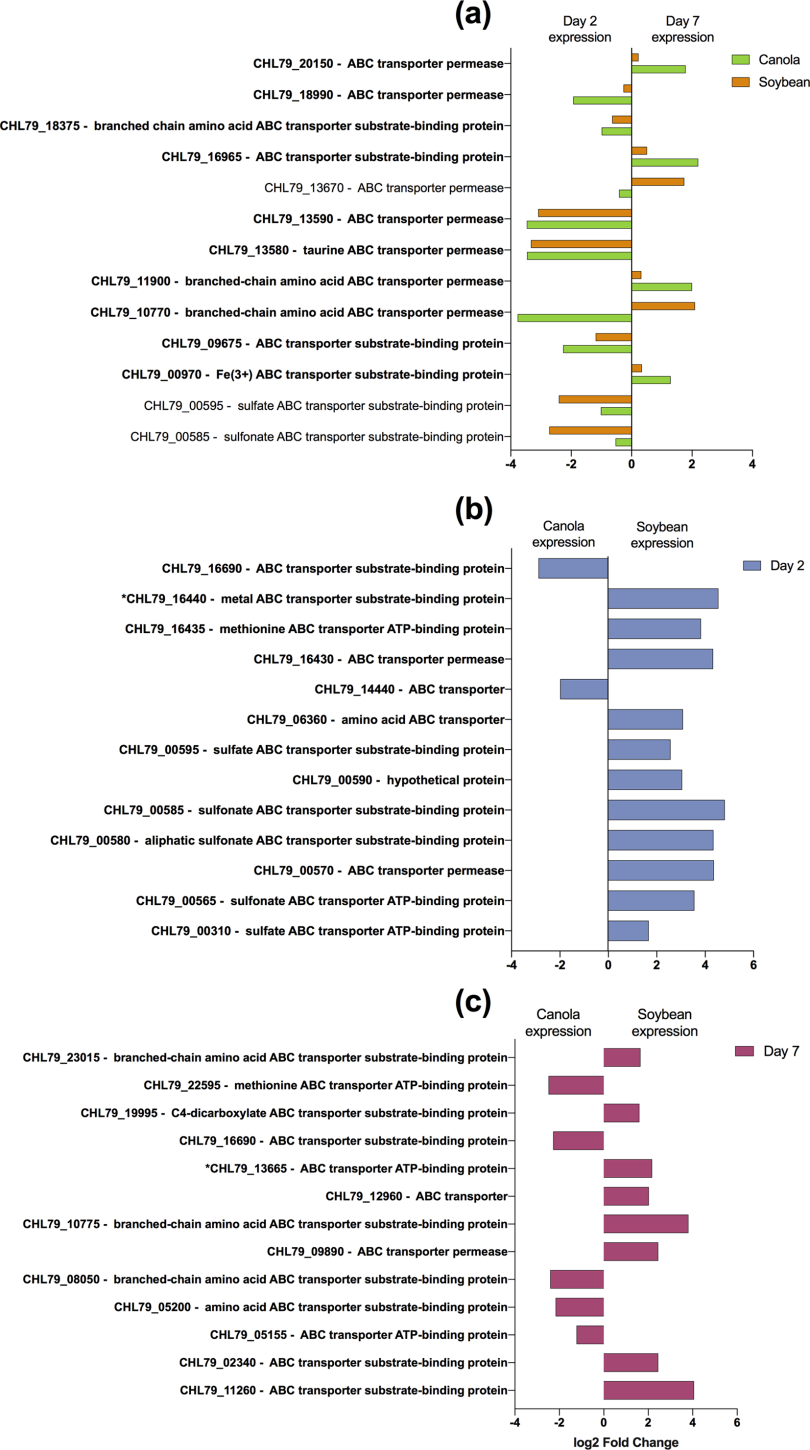
Specific sigDE cellular transporter transcripts of root-attached RAY209 cells. Locus tags with an asterisk indicate the start of a predicted gene operon. SigDE *P*adj <0.05. (a) Differential expression (DE) of specific transcripts between early (day 2) and sustained (day 7) colonization for both canola and soybean; (b) DE of specific transcripts in either canola or soybean during early colonization; (c) DE of specific transcripts in either canola or soybean during sustained colonization.

GO annotation of the 132 shared root-attached transcripts reveals that most of the cellular activities induced during RAY209 interaction with either plant host, at both early and sustained stages of colonization, fall into categories of biological process (BP) and molecular function (MF), and to a lesser extent involve the category of cellular component (CC) (Fig. 4). These categories include multiple biosynthetic processes, complex transcriptional regulation and general stress responses. Importantly, hydrolase activity, metal ion binding and membrane transport and/or remodelling (integral component membrane) were up-regulated activities, as has been demonstrated previously for *Herbaspirillum* colonization of maize [27]. The transcriptional activities of several putative ABC transporters were further examined for specific up- or down-regulation given that ABC transporters play an important role in the catabolic growth and activities of bacterial cells, which could provide insights into the nature of catabolites present in a specific environment (Fig. 5). Specific ABC transporters are similarly expressed by both canola and soybean root-attached RAY209 cells, and during the same stage of colonization, i.e. CHL79_13590, CHL79_13580, CHL79_00585 and CHL79_00595 (annotated as being related to general and sulfate/sulfonate transport) are each abundant during early colonization (D2) of either plant root (Fig. 5a). However, while there are multiple ABC transporter transcripts that share similar patterns of expression between plant types and across time, there is often a difference in the fold-change in expression of a given transcript (i.e. CHL79_20150 and CHL79_18990). It is possible that this indicates a fine-tuned response from RAY209 when initially adapting to/establishing colonization of unique plant-root environments; however, such sensitive detection is likely beyond the capacity of this dataset. Nonetheless, as demonstrated by Fig. 5(b, c), specific transcripts experience considerable difference in the direction of fold-change in expression between plant types, with genes annotated as being related to metal, methionine, sulfonate and sulfate transport experiencing considerable increase in expression during early colonization of soybean relative to canola (Fig. 5b). Following sustained colonization, there is differentiation in the expression of specific transcripts with seemingly shared or overlapping function between plant types, for example with branched-chain amino acid ABC transporters CHL79_23015 (increased abundance in soybean) and CHL79_08050 (increased abundance in canola) (Fig. 5c). This observation demonstrates a further adaptation to the respective plant environments over time, but cannot be prescriptive of specific plant-root conditions. Instead, this data supports the need for future studies to profile and correlate metabolite exudates with root colonization ability.

### Transcriptional changes in plant hosts colonized by RAY209

**Fig. 6.**
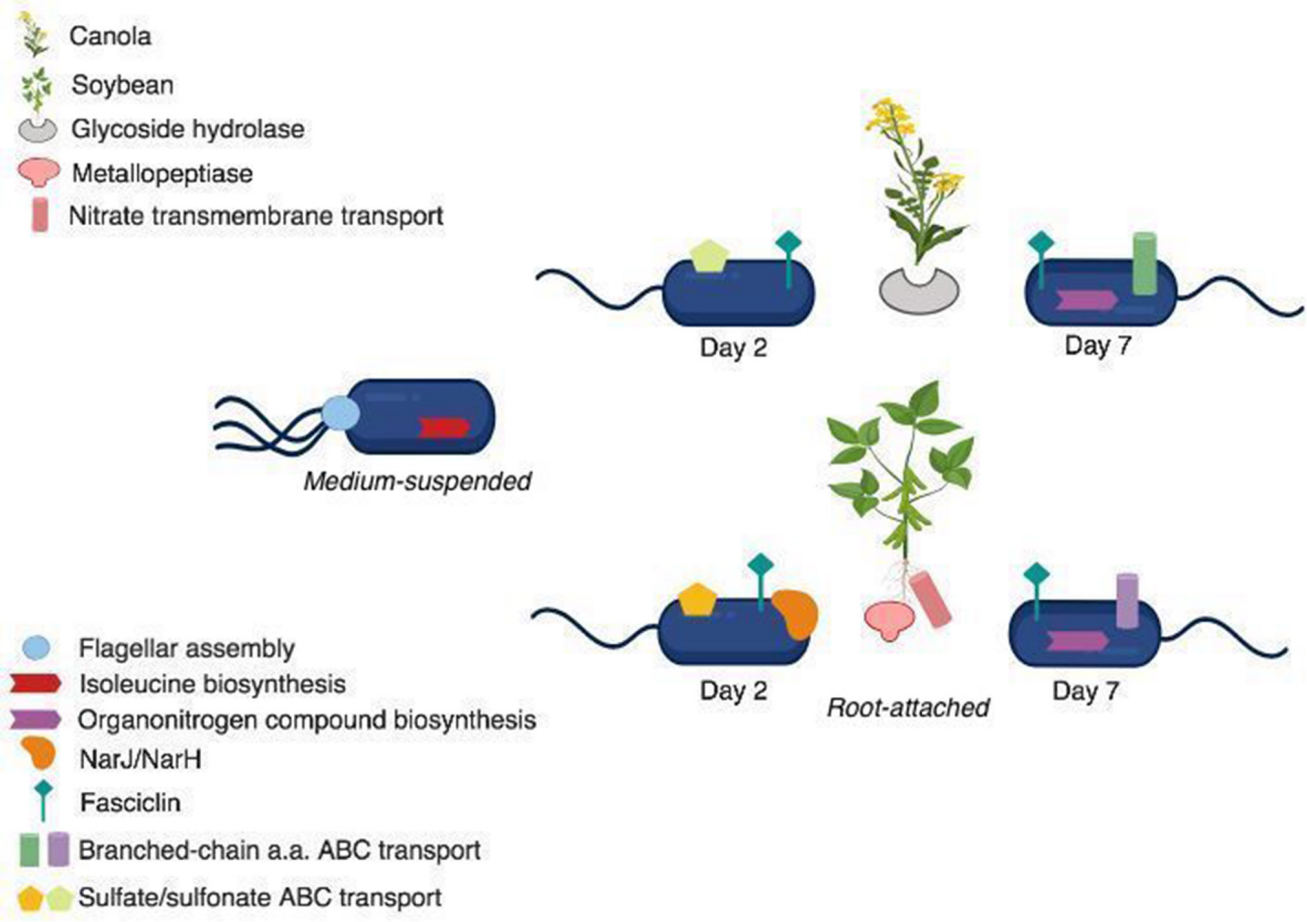
Schematic of highly abundant transcripts and processes during early (day 2) and sustained (day 7) RAY209 colonization of canola or soybean. Transcripts abundant in plant hosts experiencing RAY209 colonization are also indicated.

The co-isolation of plant mRNA alongside extracted bacterial mRNA was unavoidable; thus, a brief analysis was performed to highlight genes from each plant type that were sigDE over the length of the experiment. For instance, a canola glycoside hydrolase (BnaC08g32030D-1) with similarity to a pectin family of hydrolases was increased 4.8-fold during early colonization (D2) by RAY209 relative to sustained colonization (D7) (Fig. 6). Examination of the soybean transcriptional response to putative colonization by RAY209 revealed an 11.3-fold increase in abundance of a metallopeptidase (M10) gene (GLYMA_01G036900; *P*adj 1.70×10^−8^) during early colonization relative to sustained colonization. This class of metallopeptidases function as extracellular proteases that have a role in degrading and remodelling extracellular matrices [28]. During early exposure to RAY209, soybean plants exhibited a threefold increase in abundance of another potential cell wall remodelling gene GLYMA_01G137700 (*P*adj=0.009), which shares homology with pectin lyases, and up-regulated GLYMA_10G016500, which is a putative transcriptional regulator of unknown function. Following sustained RAY209 colonization (D7), soybean gene GLYMA_01G200100, encoding a transmembrane transport protein with homology to nitrate transporters, was present at a 22-fold greater level (*P*adj=0.0005) relative to early colonization (Fig. 6).

## Discussion

Our experimental design provided the opportunity to study the changes in transcript abundance related to the establishment of root attachment and colonization by a PGPR, and allowed us to observe broad genetic changes of the maturing root-attached population during sustained colonization. Separation of the medium-suspended cells from root-attached cells relied on the known behaviour of rhizobacteria to strongly anchor to root surfaces – a process that has been well-established through studies on bacterial root attachment [4], with additional support derived from canola root attachment assays (Figs 1 and S2 and S3) and GFP staining and visualization of root-attached cells (Fig. 2). This study provides an overview of the transcriptional responses employed by RAY209 during the colonization of two distinct plant-root systems, providing important insights into the mechanistic strategies of root establishment employed by agriculturally relevant rhizobacteria.

The observed changes in RAY209 gene expression were greatest between the medium-suspended and root-attached populations on both the canola and soybean roots, while root-attached cells on both plant types experienced limited differential expression over time. However, this survey of the broad genetic changes does allude to the existence of both (i) a core generalized gene expression response to root colonization (Fig. 4) and (ii) changes in gene expression induced by the specific planthost root environment (Fig. 6).

Bacterial root colonization is initiated with cell attachment to the root surface and has been shown to involve two phases: an initial, weak, reversible phase, followed by a strong, irreversible phase [4]. While the experimental design does not allow for assessment of the transcriptional response to a colonization ‘signal’ or triggering event that prompts RAY209 cells to initiate weak or strong attachment, the binary medium-suspended and root-attached cell states profiled permit investigation into the transcriptional activities related to these distinct bacterial lifestyles. Both phases involve the use of a variety of cell-surface components, some of which are conserved in plant-associated bacteria, while others are species-specific, including flagella, pili, exopolysaccharides and specific outer-surface proteins [4]. RAY209 demonstrated increased transcription of genes related to exopolysaccharide production, as well as CHL79_06460, which shares homology to the fasciclin family of adhesins (Figs 3 and 6). Expression of this transcript increased during RAY209 interaction with both canola and soybean roots relative to medium-suspended cells; in the case of soybean, expression continued to increase throughout root colonization, ultimately demonstrating the highest fold-change among all genes with a 10-fold increase in expression from day 2 to day 7 in soybean root-attached cells. As mentioned, fasciclin cell-surface proteins have been well-studied in plants and other eukaryotes, and have been shown to play various roles in cell adhesion and subsequent cell physiology [29], despite having relatively uncharacterized roles in bacterial physiology [30]. However, a fasciclin homologue in *Sinorhizobium meliloti,* annotated as *nex*18, was shown to be expressed in root nodules, and a strain with a *nex*18 mutation resulted in a loss of ability to form nitrogen-fixing nodules [31]. Apart from this study, the fasciclin proteins remain unstudied in PGPRs; thus, our results prompt further investigation into their role in plant-root colonization.

The up-regulation of a hypothetical protein with a ferritin-like domain (CHL79_06465) in the day 7 soybean root colonizing RAY209 population hints at the potential chemical influences within the root environment, and merits further investigation into the role of iron acquisition in sustained colonization of the mature soybean root system (Fig. 3). The production of iron-sequestering siderophores by plant-root-colonizing bacteria has been well studied due to the direct and indirect plant growth-promoting benefits of sequestering environmental iron for plant use or preventing iron acquisition by deleterious soil-dwelling microbes [32].

Of further interest are the changes in expression of transcriptional regulators between soybean and canola root-colonizing populations, as these can provide insights into how bacterial colonizers use specific regulatory networks to respond to unique environmental root signals. Many of the transcriptional regulators found to be differentially expressed are classified within the families that are known to regulate the expression of catabolic gene operons, such as the AraC family of regulators, which is logical given the nature of carbon compounds exuded by plant roots and the role of root exudation in modulating bacterial growth [33, 34]. Furthermore, an uncharacterized, two-component regulatory system, CHL79_18710/CHL79_18705, had significantly higher levels of expression in the day 2 RAY209 canola root-attached population relative to the day 7 population; this system was not expressed in the corresponding soybean population (Supplementary Excel File). It is possible to postulate that this two-component signal transduction system is sensing and regulating gene expression in response to an environmental cue specific to early developing canola root systems, which highlights the value of RNA-seq datasets in generating future hypothesis-driven research to identify new plant–microbe signalling networks.

Similar to other studies [35], the increase in expression of several predicted ABC transporter genes during root colonization was observed (Fig. 5). Many of the up-regulated ABC transporter genes are predicted to encode amino acid uptake transporters, serving the role of scavenging amino acids in plant-root exudates for RAY209 biosynthetic purposes and as sources for carbon catabolism. The diversity of transporters that experienced sigDE during colonization highlights RAY209 as a versatile PGPR that is genetically equipped to adapt to multiple plant hosts and the changing carbon exudates that result from host plant and root system maturation.

Many sigDE genes (i.e. >2 log_2_, *P*adj <0.05) are conserved hypothetical genes or genes with domains of unknown function (DUFs). For example, comparison between medium-suspended and root-attached cells during canola root colonization reveals the up-regulation of 18 hypothetical genes (sigDE; *P*adj <0.05) (Supplementary Excel File). In another case, the DUF3455-containing genes CHL79_03905 and CHL79_25525 were strongly increased in differential expression in RAY209 cells isolated from soybean roots at day 7, revealing 20-fold higher expression compared to cells isolated from day 2 roots (*P*adj <0.05). DUF3455 is also found within genomes of plant-associated *Bradyrhizobium* [36].

The co-extraction of plant-root mRNA provided an opportunity to explore the nature of the plant host transcriptome as it responds to RAY209 colonization. While it is important to note that plant maturation may play a role in these observed transcriptional changes, the nature of the differentially expressed genes supports previous studies and highlights common themes in the biological processes of plant–microbe interactions. For example, both canola and soybean responded to RAY209 exposure by increasing expression of transcripts related to the remodelling of the plant cell wall and extracellular matrix, including distinct glycolytic and proteolytic enzymes such as pectin lyases and extracellular metalloproteases. The induction of cell wall remodelling enzymes is thought to facilitate root development by controlling cell wall loosening and altering root architecture [37], and may further facilitate colonization by PGPRs [38]. Supporting this hypothesis, a legume pectate lyase has been shown to be necessary for successful nodulation by rhizobial symbionts [39], and other hydrolases are known to be important for rhizobium root infection [40]. Furthermore, the increased expression of a predicted nitrate transporter by soybean roots during sustained RAY209 colonization is similar to the reported activity of the PGPR *Pseudomonas nitroreducens* in *Arabidopsis thaliana,* where plant-nitrate transport gene expression is up-regulated in the presence of the PGPR to support nitrogen uptake [41].

Ultimately, the analysis of RAY209 transcriptional behaviour during early and sustained colonization of canola and interaction with soybean roots reveals that this isolate is capable of a strong motility response when medium-suspended, and increases the abundance of transcripts related to strong root-attachment and responsive nutrient uptake. The process of establishing initial root interactions from a medium-suspended state induces a stronger, arguably more complex, transcriptional response than does sustained interaction throughout root maturation. While this dataset prompts several routes of analysis worth further investigation – including PGPR transcriptional regulation in response to plant host and the role of fasciclin in PGPR activity – it also demonstrates the genomic capacity for RAY209 to adapt to the distinct root environments of two globally important food crops, which has implications for the application of RAY209 as both a canola and soybean PGPR seed inoculant.

## Full-Text

## Supplementary Data

Supplementary material 1

Supplementary material 2

## References

[1] Dunfield KE, Germida JJ (2001). Diversity of bacterial communities in the rhizosphere and root interior of field-grown genetically modified *Brassica napus*. FEMS Microbiol Ecol.

[2] Grayston SJ, Vaughan D, Jones D (1997). Rhizosphere carbon flow in trees, in comparison with annual plants: the importance of root exudation and its impact on microbial activity and nutrient availability. Appl Soil Ecol.

[3] Walker TS, Bais HP, Grotewold E, Vivanco JM (2003). Root exudation and rhizosphere biology. Plant Physiol.

[4] Wheatley RM, Poole PS (2018). Mechanisms of bacterial attachment to roots. FEMS Microbiol Rev.

[5] Glick BR (1995). The enhancement of plant growth by free-living bacteria. Can J Microbiol.

[6] Oresnik IJ, Mascarenhas L, Yost CK (2016). Does it take a community to raise a plant? A summary of the Canadian Crop Microbiome Workshop. Can J Microbiol.

[7] Checcucci A, DiCenzo GC, Bazzicalupo M, Mengoni A (2017). Trade, diplomacy, and warfare: the quest for elite rhizobia inoculant strains. Front Microbiol.

[8] Kloepper JW, Lifshitz R, Zablotowicz RM (1989). Free-living bacterial inocula for enhancing crop productivity. Trends Biotechnol.

[9] Nelson LM (2004). Plant growth promoting rhizobacteria (PGPR): prospects for new inoculants. Crop Management.

[10] Benizri E, Baudoin E, Guckert A (2001). Root colonization by inoculated plant growth-promoting rhizobacteria. Biocontrol Sci Technol.

[11] Fitzpatrick CR, Copeland J, Wang PW, Guttman DS, Kotanen PM (2018). Assembly and ecological function of the root microbiome across angiosperm plant species. Proc Natl Acad Sci USA.

[12] Miller HJ, Henken G, van Veen JA (1989). Variation and composition of bacterial populations in the rhizospheres of maize, wheat, and grass cultivars. Can J Microbiol.

[13] Perry BJ, Bergsveinson J, Tambalo DD, Yost CK, Khan NH (2017). Complete genome sequence of *Delftia acidovorans* RAY209, a plant growth-promoting rhizobacterium for canola and soybean. Genome Announc.

[14] Shahbandeh M (2020). Worldwide Oilseed Production in 2019-2020, by Type.

[15] Ng KSH (2011). Construction of green fluorescent protein plasmids for labelling of herbicide-degrading Delftia acidovorans MC1071. PhD Thesis.

[16] Holmes A, Birse L, Jackson RW, Holden NJ (2014). An optimized method for the extraction of bacterial mRNA from plant roots infected with *Escherichia coli* O157:H7. Front Microbiol.

[17] Bolger AM, Lohse M, Usadel B (2014). Trimmomatic: a flexible trimmer for Illumina sequence data. Bioinformatics.

[18] Langmead B, Salzberg SL (2012). Fast gapped-read alignment with Bowtie 2. Nat Methods.

[19] Dobin A, Davis CA, Schlesinger F, Drenkow J, Zaleski C (2013). STAR: ultrafast universal RNA-seq aligner. Bioinformatics.

[20] Pertea M, Pertea GM, Antonescu CM, Chang TC, Mendell JT (2015). StringTie enables improved reconstruction of a transcriptome from RNA-seq reads. Nat Biotechnol.

[21] Pertea M, Kim D, Pertea GM, Leek JT, Salzberg SL (2016). Transcript-level expression analysis of RNA-seq experiments with HISAT, StringTie and Ballgown. Nat Protoc.

[22] Zerbino DR, Achuthan P, Akanni W, Amode MR, Barrell D (2018). Ensembl 2018. Nucleic Acids Res.

[23] Anders S, Pyl PT, Huber W (2015). HTSeq– a python framework to work with high-throughput sequencing data. Bioinformatics.

[24] Love MI, Huber W, Anders S (2014). Moderated estimation of fold change and dispersion for RNA-seq data with DESeq2. Genome Biol.

[25] Götz S, García-Gómez JM, Terol J, Williams TD, Nagaraj SH (2008). High-throughput functional annotation and data mining with the Blast2GO suite. Nucleic Acids Res.

[26] Ramachandran VK, East AK, Karunakaran R, Downie JA, Poole PS (2011). Adaptation of *Rhizobium leguminosarum* to pea, alfalfa and sugar beet rhizospheres investigated by comparative transcriptomics. Genome Biol.

[27] Balsanelli E, Tadra-Sfeir MZ, Faoro H, Pankievicz VCS, de Baura VA (2016). Molecular adaptations of *H*erbaspirillum *seropedicae* during colonization of the maize rhizosphere. Environ Microbiol.

[28] Marino G, Funk C (2012). Matrix metalloproteinases in plants: a brief overview. Physiol Plant.

[29] Seifert G (2018). Fascinating fasciclins: a surprisingly widespread family of proteins that mediate interactions between the cell exterior and the cell surface. Int J Mol Sci.

[30] Moody RG, Williamson MP (2013). Structure and function of a bacterial fasciclin I domain protein elucidates function of related cell adhesion proteins such as TGFBIp and periostin. FEBS Open Bio.

[31] Oke V, Long SR (1999). Bacterial genes induced within the nodule during the *Rhizobium*-legume symbiosis. Mol Microbiol.

[32] Jin CW, Ye YQ, Zheng SJ (2014). An underground tale: contribution of microbial activity to plant iron acquisition via ecological processes. Ann Bot.

[33] Hartmann A, Schmid M, van Tuinen D, Berg G (2009). Plant-driven selection of microbes. Plant Soil.

[34] Hu L, Robert CAM, Cadot S, Zhang X, Ye M (2018). Root exudate metabolites drive plant-soil feedbacks on growth and defense by shaping the rhizosphere microbiota. Nat Commun.

[35] Pankievicz VCS, Camilios-Neto D, Bonato P, Balsanelli E, Tadra-Sfeir MZ (2016). RNA-seq transcriptional profiling of *Herbaspirillum seropedicae* colonizing wheat (*Triticum aestivum*) roots. Plant Mol Biol.

[36] Lu S, Wang J, Chitsaz F, Derbyshire MK, Geer RC (2020). CDD/SPARCLE: the conserved domain database in 2020. Nucleic Acids Res.

[37] Xu L, Zhang W, He X, Liu M, Zhang K (2014). Functional characterization of cotton genes responsive to *Verticillium dahliae* through bioinformatics and reverse genetics strategies. J Exp Bot.

[38] Vacheron J, Desbrosses G, Bouffaud M-L, Touraine B, Moënne-Loccoz Y (2013). Plant growth-promoting rhizobacteria and root system functioning. Front Plant Sci.

[39] Xie F, Murray JD, Kim J, Heckmann AB, Edwards A (2012). Legume pectate lyase required for root infection by rhizobia. Proc Natl Acad Sci USA.

[40] Robledo M, Jiménez-Zurdo JI, Velázquez E, Trujillo ME, Zurdo-Piñeiro JL (2008). Rhizobium cellulase CelC2 is essential for primary symbiotic infection of legume host roots. Proc Natl Acad Sci USA.

[41] Trinh CS, Lee H, Lee WJ, Lee SJ, Chung N (2018). Evaluation of the plant growth-promoting activity of *Pseudomonas nitroreducens* in *Arabidopsis thaliana* and *Lactuca sativa*. Plant Cell Rep.

