# Supplementary material for: Transcriptomics reveal core activities of the plant growth-promoting bacterium Delftia acidovorans RAY209 during interaction with canola and soybean roots
Source: Microb Genom. 2020 Nov 5;6(11):mgen000462. doi: 10.1099/mgen.0.000462 (PMC7725335; doi:10.1099/mgen.0.000462)
Supplement: Supplementary material 1 [file mgen-6-462-s001.pdf]

Supplementary Material

1. RAY209 canola root-attachment assay

To confirm the colonization of roots by RAY209, sterilized canola seeds were individually placed in polystyrene culture tubes (Fisherbrand) containing 10 mL of 0.4% water agar (day 0). Seeds were inoculated with 1.0×10^7 CFU of RAY209 per seed and grown in a phytotron (Conviron PGR15) with a day/night cycle of 16/8 h and 22/18°C. On day 24, roots were examined for turbidity (indicating colonization) (**Figure S1A-B**). Roots were then harvested, washed twice with 20 mL of sterile water, vortexed for 30 sec, placed on TSA plates, and incubated for 48 h at 28°C to observe root-associated RAY209 growth (**Figure S1C-D**).

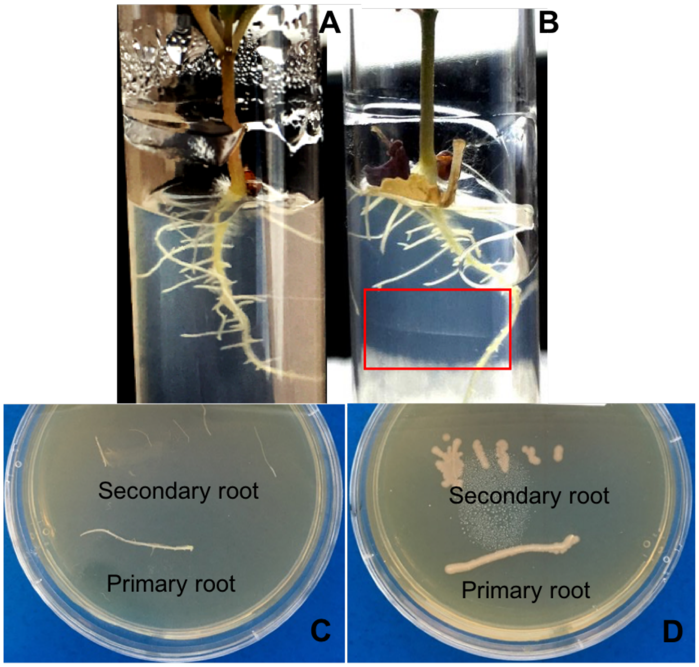

Figure S1. Canola root colonization by *Delftia acidovorans* RAY209. (**A**) and (**C**) = control (uninoculated); (**B**) and (**D**) = RAY209 inoculation. Comparison of **A** and **B** demonstrates an increase in turbidity surrounding the developing root after 24 days with RAY209 inoculation (red box). Comparison of **C** and **D** demonstrates the strength of RAY209 colonization on primary and secondary roots after 24 days.

2. Plant growth-promoting behaviour of RAY209

To monitor the effect of *Delftia acidovorans* RAY209 on root development, canola seeds were surface sterilized by submerging in 2% NaClO for 5 min and rinsed with sterile water several times. Three seeds were placed in CYG germination pouches (Mega International, Newport, MN) with 0.5× Hoagland's Solution (Sigma-Aldrich) and inoculated with 1.0×10^5 CFU of RAY209 per seed by pipetting (day 0). For effective germination and to maintain sterility, all pouches were covered with tinfoil and placed in a phytotron (Conviron PGR15) with a day/night cycle of 16/8 h and 22/18° C. Tinfoil was removed on day 4 and plants were thinned to two plants per pouch. Images of plant development were taken on day 8 (**Figure S2A-B**). Plants were harvested on day

15 and the number of lateral roots counted, and root/shoot lengths measured and weighed (**Table S1**). Prior to weighing, root/shoot material was dried at 37 °C for 4 days. The number of root hairs on developing primary roots with and without RAY209 inoculation was examined after 36 days by submerging roots in water and observing at 100× magnification (**Figure S2C-D**).

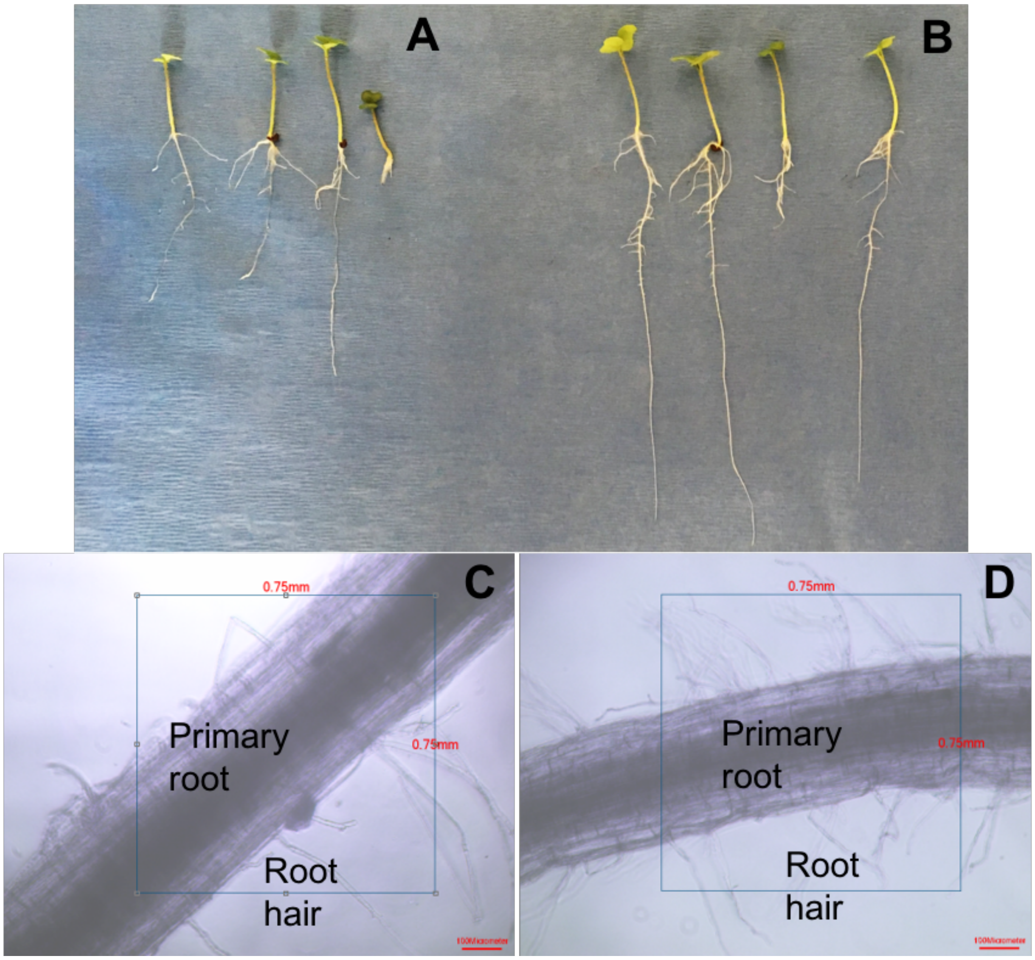

Figure S2. Effect of *Delftia acidovorans* RAY209 on canola root development. (**A**) and (**C**) = control (uninoculated); (**B**) and (**D**) = RAY209 inoculation. **A** and **B** compare differences in root length between treatments. Comparison of **C** and **D** demonstrates an increase in number of root hairs on developing primary root with RAY209 inoculation (**D**) after 36 days. Roots were submerged in water and observed at 100× magnification. Scale bars = 100 μm.

Table S1. Effect of *Delftia acidovorans* RAY209 on canola root development at day 15.

	Control	RAY209
**Dry root weight (mg)**	6.3 ± 0.9 ^{a}	7.6 ± 1.5 ^{b}
**Dry shoot weight (mg)**	17.7 ± 4.6 ^{a}	23.3 ± 3.4 ^{b}
**# of lateral roots**	71.9 ± 24.5 ^{a}	88.7 ± 12.9 ^{b}
**Root length (cm)**	22.9 ± 5.0 ^{a}	23.6 ± 3.2 ^{a}
**Shoot length (cm)**	6.6 ± 1.1 ^{a}	7.2 ± 0.6 ^{a}

Results are expressed as mean ± standard deviation (n=12 for each treatment). Different letters indicate a significant difference among treatments ($p < 0.05$).

3. Hydroponic growth system

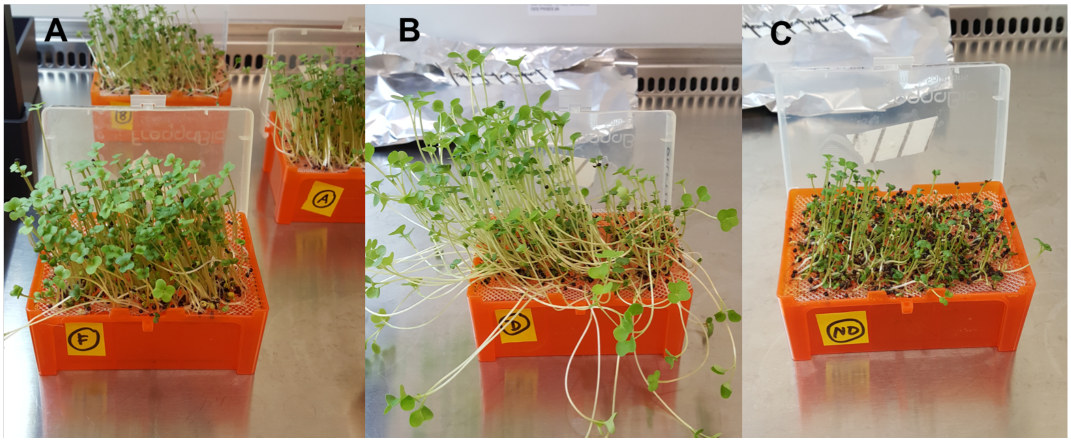

Figure S3. **Experimental growth system of canola plants.** (**A**) RAY209-inoculated plants at day 2; (**B**) RAY209-inoculated plants at day 7; (**C**) uninoculated plants at day 7.

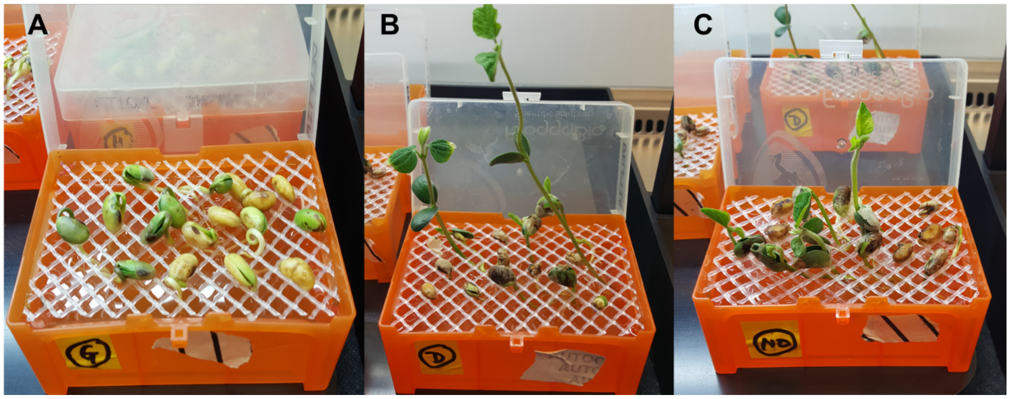

Figure S4. **Experimental growth system of soybean plants.** (**A**) RAY209-inoculated plants at day 2; (**B**) RAY209-inoculated plants at day 7; (**C**) uninoculated plants at day 7.

4. Validating strongly root-attached RAY209 isolation strategies from canola roots

Canola roots were harvested and plated as previously described in **Materials and Methods.** Homogenization and low-intensity sonication were tested for the ability to release root-attached RAY209 while preserving cell viability (**Figure S5**). A subset of processed roots (as previously described in main text) were weighed and submerged in 500 μL PBS, then bath-sonicated using the Low power setting of a Bioruptor Standard UCD-200 (Diagenode) for 5 min, alternating 30 sec on/off [1]. Cell counts were obtained as previously described.

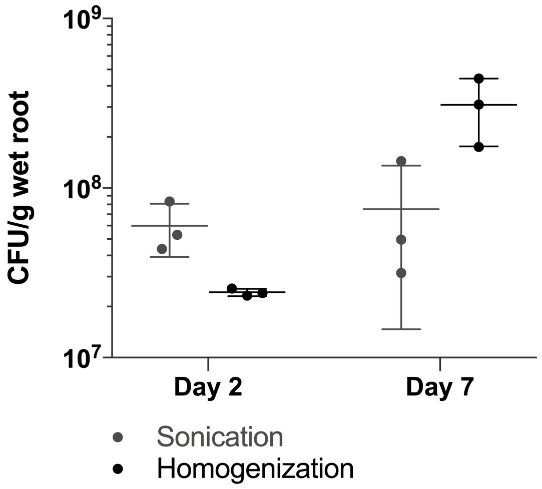

Figure S5. Validation of method for releasing root-attached RAY209 cells from canola roots. Low-intensity sonication (grey) and homogenization (black) methods were tested for the ability to release root-attached RAY209 cells from the root surface. Sonication was ineffective, as mean cell counts did not differ significantly between days 2 and 7 ($p_{two\ tail} = 0.70$). Homogenization was sufficiently effective, as a significant log_{10}-fold increase in mean root-attached RAY209 cells was observed ($p_{one\ tail} = 0.03$); n = 3 replicates.

5. RNA-seq experimental outcome

Table S2. Number of rRNA-depleted reads mapped to RAY209 and respective plant genomes.

Host	Rep.	"M" RAY209 [a]		"R" RAY209 [b]		Plant mRNA [c]	
		Day 2	*Day 7*	*Day 2*	*Day 7*	*Day 2*	*Day 7*
Canola	R1	8,684,900	6,829,641	637,630	1,904,801	16,909,580	10,494,654
	R2	7,643,694	5,954,007	792,439	1,690,933	18,002,240	9,602,052
	R3	7,407,302	6,802,323	650,666	490,109	8,950,788	10,077,618
Soybean	R1	8,487,558	8,463,258	471,852	506,971	14,828,462	14,364,480
	R2	10,209,623	7,067,879	1,016,368	696,316	1,337,766	5,651,864
	R3	7,328,668	7,961,340	1,130,917	822,095	1,816,082	3,142,692

[a] Reads from medium-suspended (“M”) mRNA samples; [b] Reads from root-attached (“R”) mRNA samples; [c] Reads mapped to respective plant genomes from root-attached (“R”) mRNA samples.

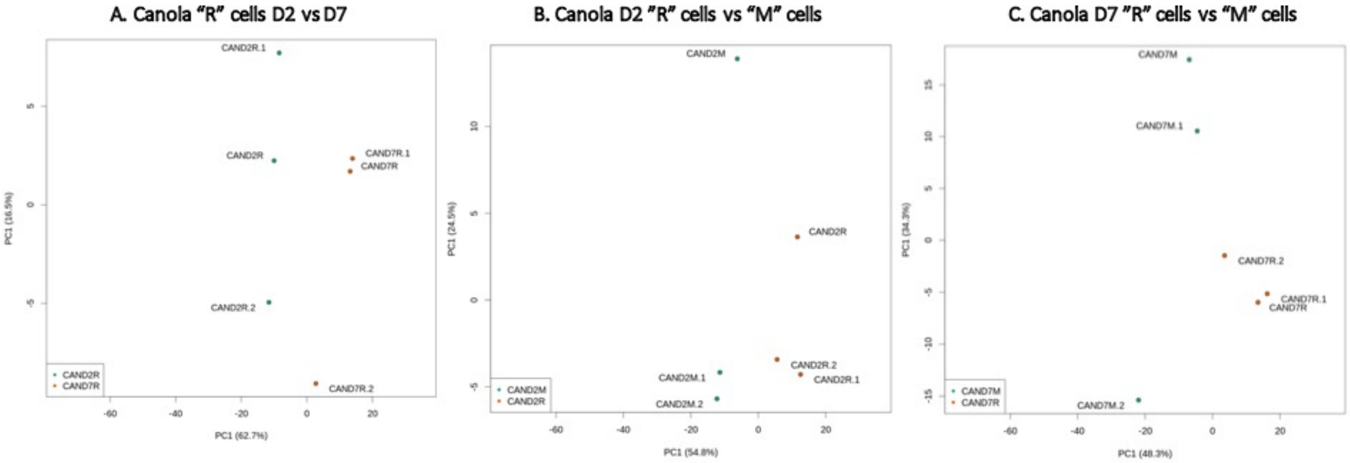

Figure S6. PCA plot of RAY209-canola expression profiles (DESeq2 regularized log transformations of count data). Comparisons of samples made are described in headings (**A** – **C**). D2 = day 2 and D7 = day 7; “M” = medium-suspended samples and “R” = root-attached samples.

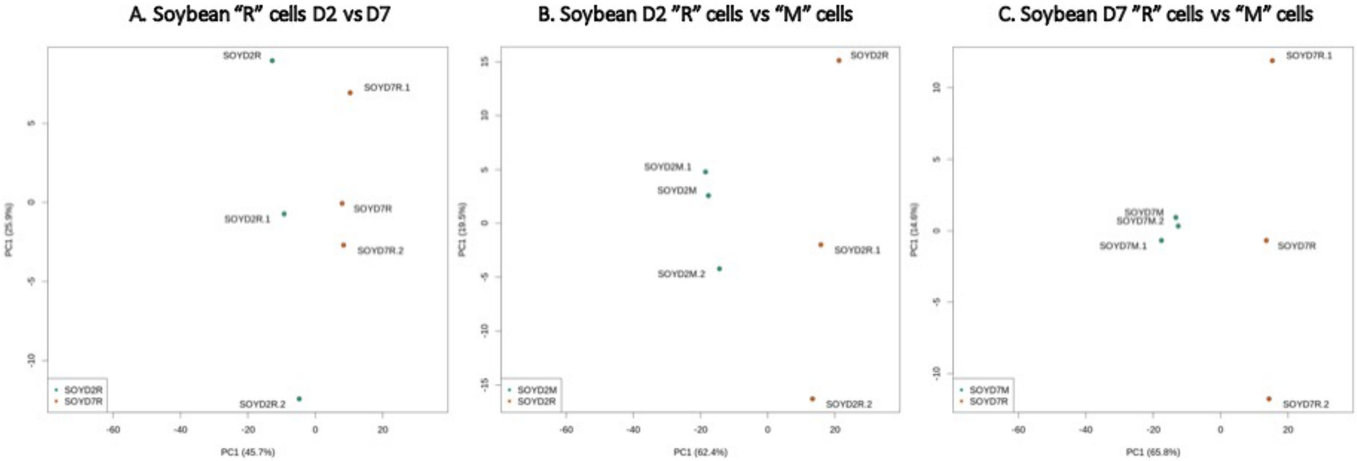

Figure S7. **PCA plot of RAY209-soybean expression profiles (DESeq2 regularized log transformations of count data).** Comparisons of samples made are described in headings (**A** – **C**). D2 = day 2 and D7 = day 7; “M” = medium-suspended samples and “R” = root-attached samples.

6. Dissolved oxygen concentration measurements

To ensure adequate oxygenation within the liquid plant growth medium, dissolved oxygen concentrations were measured at canola roots with a YSI meter prior to root harvesting on day 2 and day 7 (**Figure S8**). Before each measurement, the probe was sterilized in a 2% NaClO solution

for 3 min and rinsed with sterile water. Care was taken to not disturb the liquid growth medium during data collection.

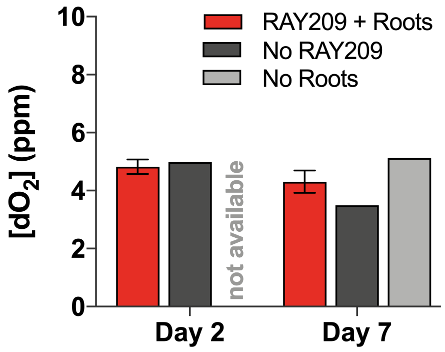

Figure S8. Average dissolved oxygen concentration around canola roots. Dissolved oxygen (dO_2) at the roots remained consistent ($p_{two\ tail} = 0.12$) throughout the week-long growth period, averaging 4.8 ppm at day 2 and 4.3 ppm by day 7 (n = 3 replicates).

6. References

1. **Richter-Heitmann T, Eickhorst T, Knauth S, Friedrich MW, Schmidt H**. Evaluation of strategies to separate root-associated microbial communities: a crucial choice in rhizobiome research. *Front Microbiol* 2016;7:773.
